# Real-Life Comparison of Diagnostic Yield and Sample Adequacy of 22 G and 25 G EBUS-TBNB Needles: A Retrospective Study

**DOI:** 10.3390/jcm14051637

**Published:** 2025-02-28

**Authors:** Filippo Lanfranchi, George Kalak, Gioele Castelli, Laura Mancino, Gabriele Foltran, Alberto Pavan, Lorenzo Ciarrocchi, Licia Laurino, Lucio Michieletto

**Affiliations:** 1 Respiratory Disease Unit, Department of Cardiac Thoracic and Vascular Sciences, Ospedale dell’Angelo, 30174 Venice, Italy; 2 Department of Medicine, Pulmonary Institute, Shaare Zedek Medical Center, Faculty of Medicine, Hebrew University of Jerusalem, Jerusalem 9103102, Israel; 3 Respiratory Disease Unit, Department of Cardiac, Thoracic, Vascular Sciences and Public Health, University of Padova, Via Giustiniani 2, 35128 Padova, Italy; 4 Pathology Unit, Ospedale dell’Angelo, 30174 Venice, Italy

**Keywords:** EBUS, Franseen needle, TBNA, TBNB

## Abstract

**Background/Objectives**: EBUS-TBNA is a safe and minimally invasive procedure to evaluate hilar and mediastinal lymph nodes (LNs). The Franseen needle provides a transbronchial needle biopsy (TBNB). Various needle sizes are available. In the literature, diagnostic yield (DY) and sample adequacy (SA) between needle sizes are still being debated. **Methods**: In total, 88 patients with lymphadenopathy were consecutively enrolled from June to December 2021. Chest CT and PET/CT scans were performed. Dimension at imaging and EBUS and the standardized uptake value (SUV) were recorded. EBUS-TBNB was performed with 22 G or 25 G needle sizes. DY for cancer and SA for predictive markers were evaluated. Overall DY (ODY) was also evaluated. **Results**: A 22 G needle was used in 51 patients and a 25 G needle was used in 37 patients with no differences in sex and age. The 22 G population presented a larger median dimension of LN both at imaging and EBUS compared to the 25 G population. Median LN SUV was higher in the 22 G population. Notably, 70 out of 88 patients had LNs suspicious for malignancy, which was higher in the 22 G group compared to the 25 G group (n = 46, 90% vs. n = 24, 65%; *p* = 0.004). DY for cancer was similar in both groups (84% for 22 G; 91% for 25 G). Also, SA for predictive markers was similar. ODY values were 78% and 92%, respectively, for the 22 G and 25 G needles. **Conclusions**: The 25 G needle has a higher DY (even if not statistically significant) and SA for predictive markers similar to the 22 G needle; further studies are necessary to evaluate if 25 G is comparable to the 22 G needle.

## 1. Introduction

Hilar and mediastinal adenopathy refer to the enlargement of lymph nodes located in the hilar and mediastinal regions of the chest, respectively. The causes of hilar and mediastinal adenopathy are diverse and can include infectious diseases (e.g., tuberculosis and histoplasmosis), inflammatory conditions (e.g., sarcoidosis), and malignancies (e.g., lymphoma and metastatic cancer). The evaluation of these conditions often involves imaging studies and may require tissue sampling through procedures like endoscopic ultrasound (EUS) or endobronchial ultrasound (EBUS)-guided fine-needle aspiration (FNA) to obtain a definitive diagnosis.

Endobronchial ultrasound transbronchial needle aspiration (EBUS-TBNA) became the standard of choice for the diagnosis and staging of pulmonary cancers, but also for the diagnosis of metastatic disease and adenopathy, allowing and facilitating the diagnosis of lymphomas, tuberculosis, and sarcoidosis more than cancer [1,2,3].

EBUS-TBNA is performed using a bronchoscope with a linear ultrasound transducer at the tip, which allows real-time visualization of the needle as it passes through the bronchial wall to sample the target. This real-time guidance significantly enhances the accuracy of the procedure compared to traditional TBNA, which lacks ultrasound visualization [4]. The utility of EBUS-TBNA extends beyond lung cancer staging to the diagnosis of various thoracic conditions. This technique is particularly valuable in the evaluation of mediastinal and hilar lymphadenopathy, where it has demonstrated high diagnostic accuracy and safety.

In fact, the diagnostic accuracy of EBUS-TBNA for mediastinal staging has been shown to be comparable to mediastinoscopy but with the benefit of a superior safety profile [3,5,6,7,8,9,10].

Because of the innovations in cancer therapies, which need additional mutational analysis such as next-generation sequencing (NGS) compared to conventional immunohistochemistry (IHC) such as programmed-death ligand 1 (PDL-1) but also, for example, epidermal growth factor receptor analysis (EGFR), the demand for more tissue and histological samples is continuously increasing, even in EBUS-TBNA procedures; in fact, the request for DNA analysis of the tissue sampled is followed by the need to obtain more tissue than which can be obtained with traditional TBNA needles, even if there are no clear indications of if a larger TBNA needle may result in augmented DY and SA [11,12,13,14,15,16,17,18,19,20,21].

In fact, The American College of Chest Physicians recommends that either 21 or 22 G needle sizes are acceptable options, as there is no significant difference in diagnostic yield for malignancy between the two sizes [1].

The choice of needle size should be determined by the operator based on factors such as the location and vascularity of the lymph node. Some studies suggest that smaller needles might reduce blood contamination when sampling more vascular nodes, while larger needles might provide higher cytologic sensitivity [1]. However, the overall diagnostic yield does not significantly differ between the 21- and 22-gauge needles, making both sizes viable options for clinical practice.

A common limitation of EBUS-TBNA is the inadequacy of the sample obtained for genomic profiling or for histological assessment when tissue architecture is necessary such as in lymphoproliferative disorders and granulomatous inflammation [22,23]. Also, tumor tissue in the form of core biopsy samples is often needed for enrollment in cancer treatment trials [24], and fine-needle aspirate (FNA) samples alone are inadequate.

Gastroenterologists with prior experience in the use of Franseen needles (or crown-cut needles) with endoscopic ultrasound have reported high yield for core biopsies without adverse events [25], moving from fine-needle aspirate (FNA) to fine-needle biopsy (FNB). In gastroenterology, FNB uses a slightly larger needle designed to obtain core tissue samples, which preserve the histological architecture. This technique was developed to overcome the limitations of FNA, particularly in cases where a larger tissue sample is needed for a definitive diagnosis. FNB has been shown to provide better tissue acquisition and diagnostic yield compared to FNA, especially for subepithelial lesions (SELs) and pancreatic masses [26,27,28].

The American Gastroenterological Association (AGA) and the American College of Gastroenterology (ACG) have noted that FNB may require fewer needle passes and provide more adequate samples for histological and immunohistochemical analysis [27,28], and while both FNA and FNB are valuable tools in gastroenterology, FNB is often preferred when a larger tissue sample is required for histological examination.

**Figure 1:**
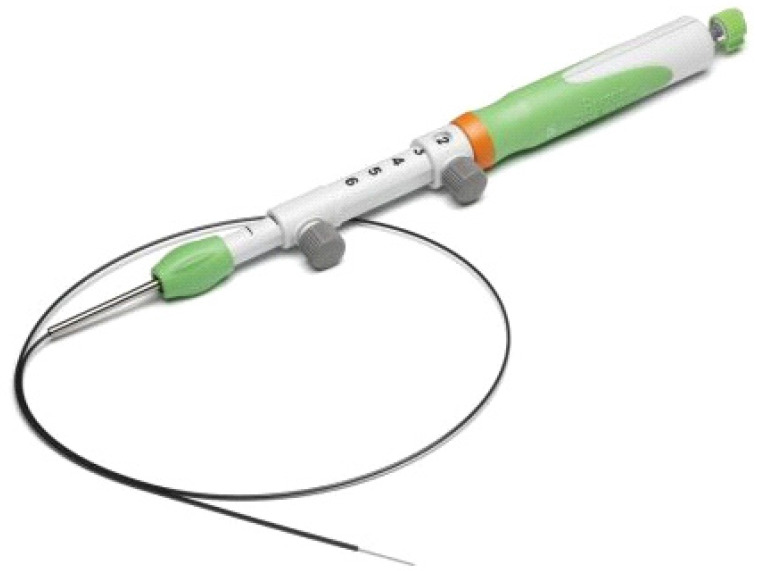
Acquire Pulmonary TBNB needle (reprinted with permission from Boston Scientific).

**Figure 2:**
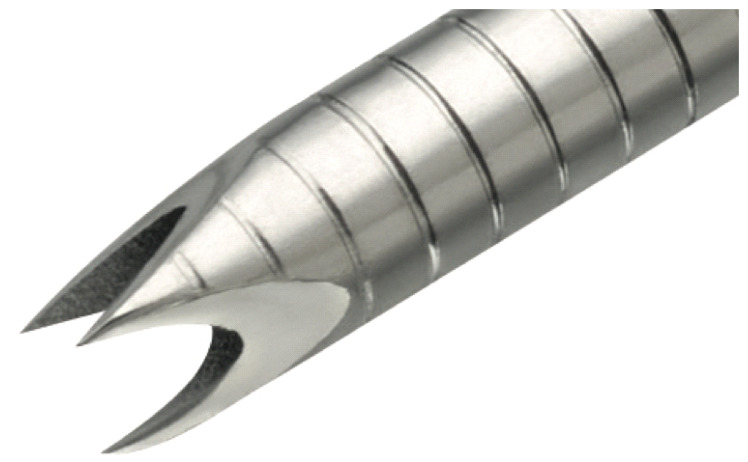
Acquire Pulmonary distal tip, i.e., the “Franseen needle tip” (reprinted with permission from Boston Scientific).

In fact, studies have shown that, compared with the fine-needle aspirate (FNA), the fine-needle biopsy (FNB) samples obtained with Franseen needles were histologically superior and required fewer passes [25,29]. Recently, a needle with a similar tip design was introduced for endobronchial use in 22 G and 25 G sizes (Acquire Pulmonary, Boston Scientific Co., Natick, MA, USA). The design of the needle tip claims the acquisition of a transbronchial needle biopsy (TBNB) specimen in the form of a tissue core (Figure 1 and Figure 2).

In the literature, DY and SA between needle sizes are still being debated [14,16,18,30]; in addition, data from novel TBNB needles are poor [31,32,33].

### Aim of the Study

This study aims to evaluate and compare the DY and SA of the 22 G and 25 G Acquire Pulmonary needles for lung cancer diagnosis.

## 2. Materials and Methods

### 2.1. Study Design and Population

In this longitudinal study, we retrospectively enrolled all the patients who underwent an EBUS-TBNB procedure between June 2021 and December 2021 in the Pulmonology Unit (Ospedale dell’Angelo, AULSS3 Serenissima, Venice–Mestre, Italy). Every patient performed a computed tomography (CT) chest scan and a positron emission tomography CT (PET-CT) scan prior to the procedure. All the procedures were performed using Acquire 22 G or 25 G Franseen needles.

Demographic, LN characteristics at CT (LN dimensions) and PET scan (maximal standardized uptake value (SUV)), needle size, and number of passes were recorded. Pre-test probability of LN metastasis was also recorded (based on anamnesis, LN dimension, radiological characteristics, SUV, and chest CT scan findings) to distinguish between LNs suspicious for malignant and non-malignant disorders (i.e., granulomatous inflammation or infectious disease). Patients were grouped according to the needle size used for the sampling in two groups: 22 G and 25 G. Needle gauge selection (22 G or 25 G) was at the discretion of the bronchoscopist. Even if there are no specific criteria for needle gauge selection, in clinical practice, thinner needles were used when abnormal vascularization was observed or suspected or even when the risk of bleeding was considered to be increased.

In patients suspected of having cancer, the true-positive sample was defined as a sample positive for cancer; a true-negative sample was defined as a sample with no evidence of cancer or a sample with lymphocytes or even granulomatous inflammation.

In patients without suspicion of cancer, true-negative samples were defined as a sample negative for cancer and non-specific for benign disease (i.e., lymphocytes); these data were confirmed after thoracic surgery biopsies or, as the alternative, by following a restricted clinical and radiological follow-up (PET/CT and/or chest CT scan) and decided by a multidisciplinary thoracic oncology committee. False-negative samples were defined as the presence of cancer in subsequent diagnostic approaches.

DY was defined as the rate between true positive and the totality of patients with pre-test probability positive for lung cancer (i.e., true positive and false negative); SA is defined as the rate between samples positive for cancer and adequate for complete IHC (next-generation sequencing [NGS] included, where indicated) by pathologists and the totality of samples positive for cancer.

Finally, overall DY (ODY) is defined as the sum of true-positive and true-negative samples (i.e., lymphocytes and sarcoidosis) divided per the totality of patients, which is intended as the overall DY for every disease (benign and malignant diseases). Patients with true-negative samples were regularly followed either clinically or radiologically, and all of them were confirmed as real true-negative cases during an adequate follow-up period (at least 12 months).

Furthermore, all complications were recorded (respiratory failure, infections, pneumothorax/pneumomediastinum, hemoptysis, death, needle break, cardiovascular disorders, and hemodynamic instability).

### 2.2. EBUS-TBNB Procedure

EBUS-TBNB was performed under moderate sedation using our institution’s standard protocol. Patients were monitored during all parts of the procedure and sedation was titrated to the patient’s comfort and ability to follow commands. According to the International Association for Study of Lung Cancer (IASLC) LN map, LNs were identified and recorded [34].

A convex probe endobronchial ultrasound (EB-530 US, Fujifilm, Tokyo, Japan) was used for EBUS-TBNB. The designated lymph node was measured and sampled under direct EBUS guidance. Needle gauge selection (22 G or 25 G) was at the discretion of the bronchoscopist. The target lesion was punctured with an Acquire TBNB needle (Acquire Pulmonary, Boston Scientific Co., Natick, MA, USA), and samples were collected using the suction technique.

The suction technique was performed as follows: After identification of the target, a needle was used to puncture the LN. After the puncture, the stylet was completely removed, and a negative-pressure vacuum syringe was applied. The needle was pushed into the target LN, and back-and-forth movements were performed at least 15 times under continuous EBUS view. After each puncture, the stylet was reinserted to push the contents out, and a syringe filled with air pushed out the remaining tissue specimen. The quality of puncture specimens was based on rapid on-site evaluation (ROSE) [2], performed using a Diff-Quik stain, to guide the number and site of the next passes. All ROSE evaluations were performed by the pulmonologist team in order to guide the number of passes.

The samples obtained were processed for cytological and histological evaluation; all pathological diagnoses were performed by pathologists at the Venice–Mestre Hospital, in the Pathologic Anatomy Unit.

### 2.3. Ethics Statement

This was a retrospective study using anonymized patient data collected from electronic medical records.

The study was approved by the Ethical Committee of Padua University (number 21482; approved on 19 January 2023).

Consent for retrospective evaluation of data was waived in accordance with local legislation.

The work described has been carried out in accordance with The Code of Ethics of the World Medical Association (Declaration of Helsinki). All patients signed a written consent before the procedure.

### 2.4. Statistical Analysis

Categorical variables are expressed as absolute (n) and relative values (%) whereas continuous variables are expressed as medians and ranges. To compare 22 G and 25 G populations, the Chi-square test and Fisher’s exact test for categorical variables and Mann–Whitney U test for continuous variables were used, as appropriate.

All data were analyzed using GraphPad Prism 7.0 (GraphPad Software Inc., La Jolla, CA, USA). *p*-values < 0.05 were considered statistically significant.

## 3. Results

We identified 88 patients who underwent EBUS-TBNB sampling of mediastinal and hilar LNs using Franseen tips from June 2021 to December 2021; descriptive statistics of patients and LNs are shown in Table 1.

A 22 G needle was used in 51 patients and a 25 G need was used in 37 patients, and no statistically significant differences were observed between sex and age in the two groups (male 57% vs. 68%, *p* = 0.07; 66 vs. 72 years, *p* = 0.19).

The 22 G population presented a larger median dimension of LN both at chest CT imaging (20 vs. 15 mm, *p* = 0.001) and at EBUS view (20 vs. 15 mm, *p* = 0.005) compared to the 25 G population.

Station 7 LN was the most sampled in the overall population (49%), but no differences were found in terms of LN sampling frequency between the needles. Conversely, the median LN SUVmax was higher in the 22 G population (12.8 vs. 8.1, *p* = 0.007). No differences in the number of passes were found between the needles.

In total, 69 of 88 patients had an LN suspicious for malignancy, which was higher in the 22 G group compared to the 25 G group (n = 46, 90% vs. n = 23, 62%; *p* = 0.007). When cancer was suspected, DY was similar in both groups (84% vs. 91%) as well as SA for predictive markers (74% vs. 71%) (Table 2).

Interestingly, overall DY (ODY) tends to be higher in the 25 G group, whereas in the 22 G group, ODY tends to be slightly lower compared to DY alone.

In the 22 G group, there were 9 small-cell lung cancers (SCLCs), 19 non-SCLCs (NSCLCs) (17 adenocarcinomas and 2 squamous cell carcinoma), 3 carcinomas not otherwise specified (NOS), metastasis from extra-thoracic cancer, and 2 lymphomas. No diagnosis for sarcoidosis was obtained, even if two cases were suspected of being malignant. One sample provided lymphocytes, and its negativity was confirmed during follow-up.

In the 25 G group, there were 5 SCLCs, 11 NSCLCs (9 adenocarcinomas and 2 squamous), 4 k NOS, and 1 metastasis from colorectal cancer. No diagnosis for sarcoidosis was obtained (one case was suspected of being malignant); 13 negative samples were obtained and confirmed at subsequent clinical and radiological follow-ups.

There were no patient-related complications observed: in particular, no complications were observed in terms of pneumothorax, mediastinitis, massive hemoptysis, needle break, respiratory failure, hemodynamic instability, and cardiac adverse events.

## 4. Discussion

Direct comparison of various EBUS needles is challenging and this study presents, to our knowledge, the first comparison between Franseen needles of different sizes. Our data show that TBNB needles have a high DY, which is concordant with data described in the literature, which ranges between 82% and 93% [35], independently of needle size.

Regarding traditional TBNA needles, malignant cells were present in higher numbers in the 22 G group compared to the 25 G needle group without difference in terms of adverse events [14,15]. Conversely, other studies comparing transbronchial needle aspiration performed with 25 G and 22 G needles, such as the one by De Felice et al., showed comparable DY and SA [16]. Similarly, Sood found no differences in DY and SA in comparison between 25 G and 21 G needle [17]. However, none of these studies mentioned Franseen needle tips. In fact, the study by Oezkan et al. which compared conventional TBNA needles and Franseen tip needles revealed no differences in terms of DY; yet, it was concluded that the crown-cut needle cannot reach every LN station [32], giving our study, where every LN has been sampled without any limitation, an added value.

More recent studies compared 22 G TBNA to 22 G TBNB needles showing no difference in terms of DY, suitability for PDL1 analysis, or complications (Kramer et al. [36]), and higher DY in benign disease for TBNB yet no difference in malignant diagnosis (Aboudara et al. [37]). NGS (next-generation sequencing) adequacy was higher in TBNB samples; however, there was no clear differentiation of which needle was used first for “eased entry”.

Brown et al. [38] compared 22 G Acquire TBNB to 21 G TBNA Olympus showing equal DY. TBNB was better for malignant disorders and PDL-1 suitability. Again, difficulty was encountered in reaching some stations with the Franseen needle.

Furthermore, Balwan et al. [31] studied TBNB biopsies in 100 patients, showing safety and adequacy for histopathological diagnosis; however, no comparison between different needle sizes was conducted.

Recently, cryo-EBUS or EBUS–transbronchial mediastinal cryobiopsy (EBUS-TMC) appeared in the pulmonology field as an alternative to EBUS-TBNA. Cryo-EBUS refers to a technique known as mediastinal transbronchial cryobiopsy guided by endobronchial ultrasonography. This procedure combines the use of cryobiopsy, which involves freezing tissue samples to obtain larger and higher-quality specimens, with endobronchial ultrasound (EBUS) guidance to accurately target lesions and lymph nodes within the mediastinum.

The primary advantage of cryo-EBUS over traditional EBUS-guided transbronchial needle aspiration (EBUS-TBNA) is its ability to obtain larger tissue samples, which enhances diagnostic yield. According to a study by Salcedo Lobera et al., cryo-EBUS achieved a diagnostic performance of 94.5%, compared to 60% for EBUS-TBNA, and demonstrated a high safety profile [39]. This makes cryo-EBUS a valuable tool in the diagnosis of both malignant and benign mediastinal diseases.

Nevertheless, a recent meta-analysis by Zhenming et al. revealed that EBUS-TMC does not confer a significant advantage in diagnosing lung cancer when compared to EBUS-TBNA, whereas EBUS-TMC demonstrated a significantly higher diagnostic accuracy for lymphoma and benign diseases [40].

The differences between EBUS-TBNB and EBUS-TMC seem to be significant for which concerns lymphoproliferative disorders and benign disease but also in the materials used; in fact, in EBUS-TMC, the “way of entry” of the flexible cryoprobe is performed with a needle knife or a previous TBNA site, as seen in the works of Zhang et al. and, for example, in the work of Gonuguntla et al., respectively [40,41].

The above-mentioned studies have compared TBNA needles with cryoprobes, but none of them mentioned the Franseen needle tip; moreover, EBUS-TBNB is performed with a single needle and, according to the literature and to the most recent pieces of evidence, is not inferior to the more recent EBUS-TMC, for which concerns lung cancer diagnosing and staging.

Regarding complications, EBUS-TMC revealed adverse events (pneumothorax, pneumomediastinum, and bleeding) not encountered in EBUS-TBNB procedures [42,43,44].

Our study has some strengths. First, and in contrast to most of the published papers, we compared two different sizes of Franseen needles.

Second, we were able to use the TBNB needle on all of the different stations, without any limitations.

Interestingly, we did not encounter any clinically significant complications requiring interventions. We hypothesize that this is due to the smaller gauge size of these needles, compared to 19 G and 21 G TBNA needles, rather than the novel tip. Another possibility is that fewer passes are needed when performing TBNB where ROSE is available in the endoscopy suite which makes the procedure less traumatic.

In this regard, the use of rapid on-site evaluation (ROSE) can increase the diagnostic rate in this context. According to the American College of Chest Physicians, ROSE has been shown to decrease the number of needle passes and reduce the need for additional diagnostic procedures during endobronchial ultrasound-guided transbronchial needle aspiration (EBUS-TBNA) [1].

For example, a prospective randomized clinical trial by Oki et al. demonstrated that ROSE significantly reduced the number of punctures required (2.2 vs. 3.1 punctures; *p* < 0.001) and decreased the need for additional procedures (11% vs. 57%; *p* < 0.001) without significantly affecting the diagnostic yield for lung cancer (88% with ROSE vs. 86% without ROSE) [45].

However, other studies, including retrospective analyses by Griffin et al. [46] and Murakami et al. [47], found no significant difference in diagnostic yield between procedures performed with or without ROSE, although ROSE did reduce the number of aspirates per procedure.

While the usefulness of ROSE has been debated over past years, we noticed an increased quality of the specimen evaluated with TBNB needles, suggesting the fact that samples obtained are better than previous TBNA samples; in the literature, there are actually no data regarding the quality of ROSE performed with TBNA samples and TBNB samples.

Another interesting point is the similarity of DY between the needles in malignant diseases, but the difference in ODY is in favor of the 25 G needle; this is due to the sum of negative samples (i.e., TN) to the TP ones for malignancy or specific disorders. We hypothesize that the 25 G needle was used to sample smaller LNs as described above, so the reporting of lymphocytes was more frequent, yet not less relevant.

Moreover, the 25 G needle size had never been evaluated before for TBNB samples. In the literature, 25 G is mentioned in the gastroenterology (GE) field where, being thinner than a 22-gauge needle, it makes it easier to manipulate and potentially associated with fewer complications, such as less tissue trauma and reduced blood contamination [48,49].

In fact, in the context of endoscopic ultrasound-guided fine-needle aspiration (EUS-FNA) for pancreatic masses, studies have shown that 25-gauge needles are easier to handle and result in fewer procedure-related complications compared to 22-gauge needles, while maintaining similar diagnostic accuracy [48]. The American Society for Gastrointestinal Endoscopy also notes that smaller needles, such as the 25-gauge needle, may be preferable for certain procedures due to their flexibility and ease of use in accessing small or mobile lesions [49].

In the present study, according to GE findings, 25 G revealed a high DY without complications developing; these findings may suggest that a 25 G needle can be a valid choice for EBUS procedures like 21 G or 22 G needles.

## 5. Limitations

On the other hand, there are some limitations that should be mentioned. First, this is a single-center retrospective experience, and a limited number of patients were enrolled. However, this is also explained by the novelty of the Acquire Pulmonary needle.

Second, a possible unconscious selection bias appeared in our population; in fact, the 25 G needle was used in patients presenting with lower SUV max and smaller LN dimensions and without the suspicion of malignancy. This suggests that, in our practice, bronchoscopists decided to use the bigger needle for bigger targets. Nevertheless, these results show equal DY for 22 G and 25 G needles. Moreover, in our clinical practice, another possible explanation is that thinner needles were used when abnormal vascularization was observed or suspected, and the risk of bleeding was considered increased in order to reduce potential adverse events.

Third, even if the Franseen needle tip is adequate for diagnosing and staging lung cancer, in the present study, it seems it does not increase DY in terms of lymphoproliferative disorders and sarcoidosis, even if the sample size is too small to make definitive conclusions. These findings suggest that EBUS-TMC should be considered when the suspected disease is sarcoidosis or lymphomas, for example.

## 6. Conclusions

In conclusion, 25 G Franseen needles, although statistically insignificant, presented a slightly higher DY compared to 22 G needles, which is more evident for ODY. Furthermore, both needles presented a good SA and their use does not reveal any kind of complications.

Further studies and multi-centric evaluation for DY and SA in TBNB needles are needed to evaluate if the Franseen tip needle can be considered the standard of care for diagnosing hilar and mediastinal lymphadenopathy, independently of its size. Future studies are also required to evaluate DY and SA for lymphomas and DY for granulomatous disorders by using Franseen tips.

## Figures and Tables

**Table 1:** Descriptive statistics of patient and LN characteristics.

	Overall Population (n = 88)	22 G (n = 51)	25 G (n = 37)	*p*
Male—n (%)	57 (65)	29 (57)	28 (68)	0.07
Female—n (%)	31 (35)	22 (47)	9 (24)	
Age—yr	67.5 (46–83)	66 (46–82)	72 (48–83)	0.19
Node EBUS dimensions—mm	18 (5–50)	20 (6.5–50)	15 (5–40)	0.005
Node CT dimensions—mm	16.5 (5–61)	20 (7.5–61)	15 (5–40)	0.001
SUV at PET	11.2 (3.3–31)	12.8 (3.3–31)	8.1 (4–19)	0.007
Suspicion of malignancy—n (%)	69 (78)	46 (90)	23 (62)	0.007
TBNA passes—n	4 (1–8)	4 (1–8)	4 (2–6)	0.66
ATS Station				
4R	35 (40)	22 (47)	13 (35)	0.45
4L	7 (8)	5 (10)	2 (5)	0.45
7	43 (49)	27 (53)	16 (43)	0.47
10R	12 (14)	7 (14)	5 (14)	0.98
10L	3 (3)	2 (4)	1 (3)	0.76
11R	9 (10)	4 (8)	5 (14)	0.48
11L	8 (9)	5 (10)	3 (8)	0.78

**Table 2:** Diagnostic yield and sample adequacy in both Franseen needle sizes when malignancy is suspected and overall diagnostic yield (ODY) for all diseases.

	22 G	25 G
Diagnostic Yield (DY)	84%	91%
Sample Adequacy (SA)	74%	71%
Overall DY (ODY)	%78	%92

## Data Availability

The data that support the findings of this study are available from the corresponding author, Lanfranchi F., upon reasonable request.

## Abbreviations

The following abbreviations are used in this manuscript:

EBUS	Endobronchial ultrasound
TBNA	Transbronchial needle aspiration
NGS	Next-generation sequencing
PDL-1	Programmed-death ligand 1
DY	Diagnostic yield
SA	Sample adequacy
FNA	Fine-needle aspirate
FNB	Fine-needle biopsy
TBNB	Transbronchial needle biopsy
SUV	Standardized uptake value
ODY	Overall diagnostic yield
NSCLC	Non-small-cell lung cancer
SCLC	Small-cell lung cancer
NOS	Not otherwise specified
